# Renal-Limited Thrombotic Microangiopathy in a Patient Who Received Gemcitabine, Ramucirumab, and Pembrolizumab: A Case Report and Literature Review

**DOI:** 10.7759/cureus.53669

**Published:** 2024-02-05

**Authors:** Sumit S Patel, Hui Yi Shan

**Affiliations:** 1 Division of Nephrology and Hypertension, Department of Medicine, Keck School of Medicine, University of Southern California, Los Angeles, USA

**Keywords:** acute kidney injury (aki), cancer treatment side effects, anti-neoplastic therapy, cancer drug induced tma, thrombotic microangiopathy (tma)

## Abstract

Cancer drug-induced thrombotic microangiopathy (DITMA) is an important and serious cause of kidney disease in cancer patients. In addition to classical chemotherapy, the increasing use of targeted therapy and immunotherapy has led to more oncotherapy-associated thrombotic microangiopathy (TMA). It is important for clinicians to recognize this potentially life-threatening adverse effect and gain knowledge of the patient’s clinical course and treatment response. In this paper, we report a patient with lung cancer, who was treated with three different classes of anti-neoplastic agents, gemcitabine, ramucirumab, and pembrolizumab. This patient subsequently developed renal-limited thrombotic microangiopathy(rTMA) requiring hemodialysis. The varying features of TMA caused by these therapies were discussed. We also described the clinical course, diagnostic challenges, and management of this patient.

## Introduction

Thrombotic microangiopathy (TMA) associated with anti-neoplastic therapy is a severe complication that can disrupt cancer treatment and lead to significant morbidity and mortality. Ramucirumab, pembrolizumab, and gemcitabine are Food and Drug Administration (FDA)-approved therapies for the treatment of multiple cancers and have been independently associated with the development of TMA [1-3]. We describe a case of renal-limited TMA (rTMA) in a patient who received multi-drug therapy that included gemcitabine followed by a single dose of ramucirumab and pembrolizumab. She had a rapid and progressive decline in renal function. This case illustrated the severity of renal disease that can be associated with this multi-drug regimen. Furthermore, it detailed the diagnostic and therapeutic challenges that clinicians faced in identifying the culprit agent in a patient who was treated with combination anti-neoplastic therapy.

## Case presentation

**Figure 1:**
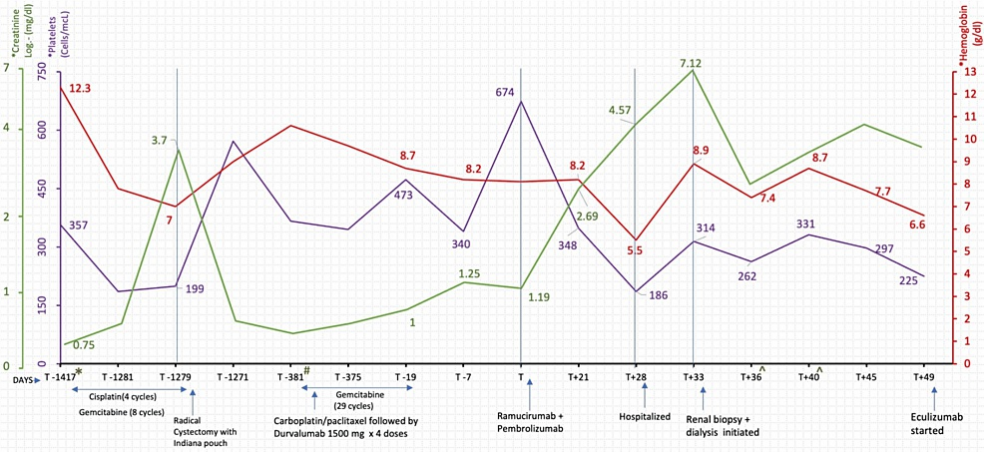
Association of timing of drug dose with hemoglobin, platelet, and creatinine. *Urothelial cancer was diagnosed. #Non-small cell lung cancer was diagnosed. ^Lactate dehydrogenase on T+36 was 366 and on T+40 was 316 (normal value: 135-214 units/L); haptoglobin on T+36 was 257 and on T+40 was 135 (normal value: 30-200 mg/dL).

A 76-year-old female with a history of high-grade urothelial cancer and stage III non-small cell lung cancer (NSCLC) was sent to the hospital for evaluation of acute kidney injury (AKI). Her urothelial cancer was successfully treated with cisplatin and gemcitabine, followed by radical cystectomy approximately three years ago. She was diagnosed with NSCLC approximately one year ago and received carboplatin/paclitaxel and radiation therapy, followed by durvalumab. The patient developed isolated oligoprogression in the left tibia, which was treated with curettage, cryoablation, and open reduction and internal fixation (ORIF). She subsequently received gemcitabine for approximately one year, with a cumulative dose of gemcitabine around 2.9 g/m^2^ (see Figure 1 for timeline). Due to cancer progression, gemcitabine was stopped, and she received one dose of ramucirumab and pembrolizumab 19 days after stopping gemcitabine. On presentation, she reported feeling fatigued with decreased appetite but denied fever, chills, or productive cough. She was taking pregabalin, acetaminophen, and tramadol at home for pain control. Vital signs showed a temperature of 35.9°C, heart rate of 80 beats per minute, blood pressure of 145/95 mmHg, oxygen saturation of 98% on room air, and weight of 49.7 kg. Physical examination was notable for bibasilar crackles on the lung examination and a lack of peripheral edema. The rest of the physical examination was unremarkable.

The patient’s laboratory tests were notable for a baseline creatinine of 0.9 mg/dL approximately three weeks before ramucirumab and pembrolizumab. After receiving the first dose of ramucirumab and pembrolizumab, serum creatinine increased from 1.2 mg/dL to 4.57 mg/dL in four weeks (Figure 1). She was also noted to have a 24-hour urine protein of 5,736 mg (Table 1). She had minimal proteinuria (50 mg/dL) on urinalysis at eight months prior to presentation. Renal ultrasound showed normal-sized kidneys without evidence of a mass, calcification, or hydronephrosis. She was started on hemodialysis for stage III oliguric AKI. AKI secondary to ramucirumab and/or pembrolizumab was considered, and both medications were discontinued.

**Table 1:** Relevant laboratory findings. PT: prothrombin time, INR: international normalized ratio, WBC: white blood cell, RBC: red blood cell, eGFR: estimated glomerular filtration rate.

Test	Result	Reference range
White blood cells (10^3^ cells/μL)	14.1	4.1-10.9
Hemoglobin (g/dL)	6.8	11.7-15.7
Platelets (10^3^ cells/μL)	220	150-400
Peripheral smear	No schistocytes seen	
PT (seconds)	14.6	12.3-14.9
INR	1.1	0.9-1.1
Sodium (mmol/L)	137	136-145
Potassium (mmol/L)	3.6	3.4-5.2
CO_2_ (mmol/L)	19	22-29
Blood urea nitrogen (mg/dL)	55	8-23
Creatinine (mg/dL)	4.9	0.51-0.95
Phosphate (mg/dL)	7.1	2.5-5.5
Total bilirubin (mg/L)	0.5	1.2
Urine nitrite	Negative	
Urine glucose (mg/dL)	50	
Urine WBC	60+	
Urine RBC	11-20	
24-hour urine protein (mg)	5,736	<0.1
Phospholipase A2 receptor antibody	Negative	
Serum protein electrophoresis with immunofixation	No abnormal bands	
Urine protein electrophoresis with immunofixation	No abnormal bands	
Kappa/Lambda ratio	2.49	0.54-3.30 (with eGFR <30)
Blood cultures	No growth over 5 days	

**Figure 2:**
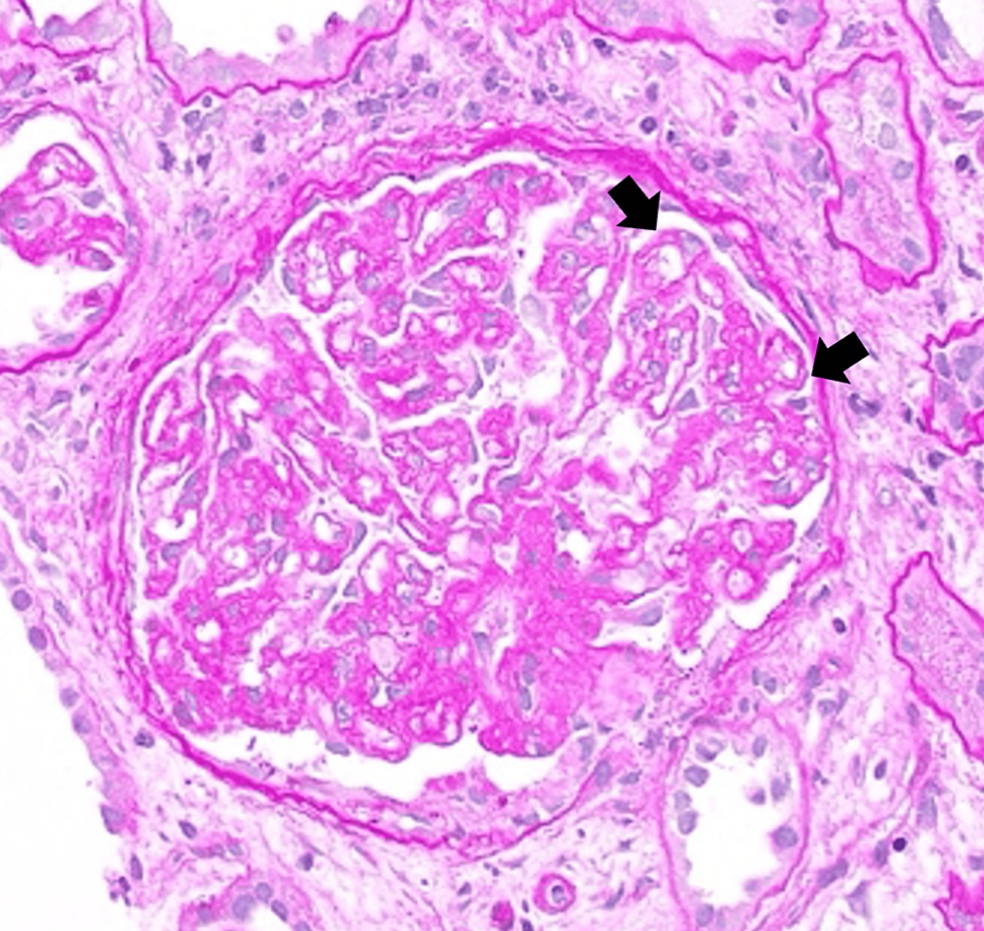
Light microscopic appearance of a glomerulus reveals global capillary wall wrinkling and double contour formation (arrows), consistent with remodeling due to thrombotic microangiopathy (periodic acid-Schiff stain; original magnification 400x).

**Figure 3:**
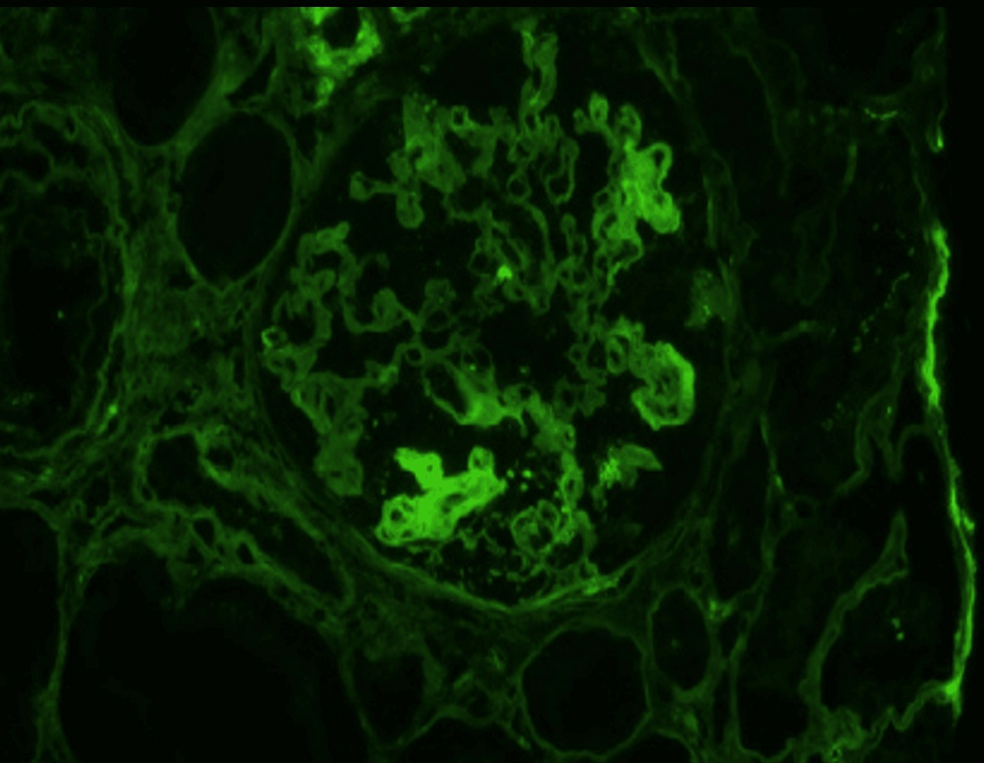
Fibrinogen stain reveals segmental staining in the capillary loops, consistent with thrombotic microangiopathy (immunofluorescence stain; original magnification 400x).

A kidney biopsy was performed, and light microscopy revealed severe arterial nephrosclerosis in a background of moderate interstitial fibrosis and tubular atrophy. Two glomeruli were present and revealed global capillary wall wrinkling with double contours and areas of mesangiolysis with acute tubular necrosis (Figure 2). The non-atrophied tubules demonstrated prominent tubular injury with loss of brush border staining and reactive nuclear changes. Immunofluorescence showed strong luminal staining for fibrinogen in arterioles and segmental staining in glomeruli (Figure 3). No glomeruli were seen on electron microscopy.

This patient had normal platelet counts and no evidence of hemolysis (normal total bilirubin, no schistocytes on peripheral smear, normal haptoglobin). A diagnosis of renal-limited thrombotic microangiopathy (rTMA) was made. We did not check ADAMTS 13 (a disintegrin and metalloprotease with thrombospondin type motifs, member 13) due to a low suspicion of thrombotic thrombocytopenic purpura. This patient had a low predictive ADAMTS 13 deficiency score (0/3 points) based on the French scoring system, and genetic evaluation for complement factor was not performed. The patient was treated with eculizumab 900 mg weekly. After three months, the patient remained hemodialysis dependent.

## Discussion

TMA is a group of disorders characterized by disseminated occlusive microvascular thrombosis, thrombocytopenia, and ischemic end-organ damage, most commonly in the kidneys and brain [4]. Various anti-neoplastic therapies have been reported to be associated with TMA. These include chemotherapeutic agents (gemcitabine, mitomycin C, and platinum-based drugs), targeted therapy (vascular endothelial growth factor (VEGF) inhibitors, tyrosine kinase inhibitors, and proteasome inhibitors), and immune checkpoint inhibitors (ICI) [1-3]. While these agents have all been reported to cause TMA, their mechanism of actions, clinical features, and disease courses differ.

Cancer drug-induced TMA is divided into two distinct categories: type I and II. The onset of type I is usually six to 12 months after starting therapy in a dose-related manner. This type appears to have permanent and irreversible kidney injury. Drugs characteristic of type I include mitomycin C, gemcitabine, and platinum salts. The pathological finding is both arteriolar and glomerular capillary thrombosis. Type II cancer drug-induced TMA occurs any time after initiation of the treatment in a dose-unrelated manner. This type has a high likelihood of recovery by stopping the drug. Drugs characteristic of type II include VEGF pathway inhibitors. Glomerular capillary thrombosis is exclusively found, but there is no arteriolar lesion in type II [5]. Our patient received therapies that consisted of gemcitabine, ramucirumab, and pembrolizumab.

Gemcitabine is a pyrimidine analog, acting as a cytotoxic agent by affecting deoxyribonucleic acid (DNA) synthesis, and it can cause TMA via both direct endothelial damage and deposition of immune complexes [1]. Gemcitabine-induced TMA can occur immediately after administration, but the median time is approximately eight months, with an average cumulative dose of around 20 g/m^2^. Concomitant use of other drugs has been shown to lower this threshold [1]. A retrospective study demonstrated 120 cases of gemcitabine-associated TMA, in which a majority of the patients presenting with systemic features of TMA were as follows: hemolytic anemia (95.6%), thrombocytopenia (74.6%), and AKI (97.4%), and dialysis was required in 27.8% of patients [6].

Ramucirumab is a human vascular endothelial growth factor receptor 2 (VEGFR2) antagonist. VEGF is produced by podocyte, and it is required for the health and maintenance of the adjacent glomerular endothelium. VEGF inhibition leads to the loss of fenestrated endothelial cells, microvascular injury, and TMA [1]. The clinical presentation of VEGF inhibition includes a new-onset or exacerbation of hypertension, proteinuria, and mainly kidney-limited TMA, with only mild anemia or thrombocytopenia. Prior case series showed that renal function, proteinuria, and blood pressure improved when the drug was withdrawn, suggesting that the processes are reversible [7].

Pembrolizumab is an immune checkpoint inhibitor that binds to programmed cell death protein 1 (PD-1) and overcomes tumor-mediated inhibition of T-cell function [3]. Most reported ICI-associated AKI is in the form of acute interstitial nephritis. However, with the increasing use of ICI in treating various cancers, few cases of ICI-associated renal limited and systemic TMAs have been reported [8]. The pathophysiology of ICI-induced TMA is not yet known, but the time from the last administration of ICI to the onset of TMA was between seven and 22 days in the reported cases. In all cases, TMA continued even after drug discontinuation [5].

Malignancy itself can also lead to TMA. Most cases of cancer-associated TMA have been reported in patients with mucin-producing adenocarcinoma and in those with disseminated malignancies [4]. Gastric carcinoma was most commonly reported in cancer-associated TMA (26.2%) followed by breast (21.4%), prostate (13.7%), and lung (9.5%) cancers [9]. Our patient developed significant deterioration in renal function approximately over three weeks after a single dose of ramucirumab and pembrolizumab. This temporal association better supports cancer drug-induced TMA, specifically type II rather than type I. In addition, our patient developed nephrotic range proteinuria and kidney-limited TMA. These findings were most consistent with VEGF inhibitor (ramucirumab)-induced TMA. However, the patient’s kidney biopsy showed evidence of both acute and chronic TMA; both arteriolar and glomerular thrombosis were present in addition to double contour of the glomerular basement membrane in a background of moderate interstitial fibrosis and tubular atrophy. With these findings, gemcitabine-induced chronic injury cannot be excluded entirely. The severe presentation and the poor renal outcome despite stopping the medication are also atypical of VEGF inhibitor-induced TMA as the renal function and proteinuria usually improve with drug withdrawal. This observation raised the question of whether the use of multi-agent therapies that can cause TMA contributes to the severity and irreversibility of this patient’s kidney injury despite stopping the medications followed by treatment with eculizumab.

## Conclusions

DITMA can be a potentially serious side effect of cancer therapies and multi-drug regimens. It is important for clinicians to become familiar with the mechanism of actions and clinical presentations of DITMA that are associated with different onco-therapeutic agents. This case is a unique example of a patient being treated with three different anti-neoplastic medications; each has been independently reported to cause TMA via a different mechanism. Whether or not the risk of developing TMA is additive when these therapies are combined is unknown. This case illustrates the severity of AKI in a patient treated with a multi-drug regimen that consisted of gemcitabine followed by ramucirumab and pembrolizumab. We recommend close monitoring for the development of DITMA when this multi-drug therapy is used.
